# Sociodemographic and health determinants of lifestyle changes during the COVID-19 pandemic in Oman

**DOI:** 10.1016/j.heliyon.2024.e40358

**Published:** 2024-11-13

**Authors:** Basma Al Yazeedi, Azza Al Marshoudi, Hajar Alufi, Mallak Al Salmi, Dakariyat Al-Sharji, Yaqoob Al Hinai

**Affiliations:** College of Nursing, Sultan Qaboos University, AlKhoud 66, Muscat, 123, Oman

**Keywords:** Coronavirus infections, Lifestyle, Obesity, Nutrition, Exercise

## Abstract

The COVID-19 pandemic preventive measures have successfully limited the spread of the infection but instituted changes in daily activities. This study examined the sociodemographic and health factors associated with lifestyle behavior changes among Omani adults during the COVID-19 pandemic. Sociodemographic factors investigated were age, gender, education, study (college versus non-college students), and marital status. A cross-sectional design was followed. We translated and tested a 20-item lifestyle behavior change during the COVID-19 pandemic questionnaire. A total of 515 responses were received with an average age of 25.7 years (SD ± 6.83). The participants' lifestyle behavior changes score mean was -2.75 (SD ± 9.08), indicating negative lifestyle change behavior. We found that lower lifestyle behavior changes scores were associated with younger age (p < .001), being single (p = .012), being a college student (p = .004), and having gained weight or were unsure about the weight change during the pandemic (p < .001). The linear regression model predicted a .19 increase in the lifestyle behavior change scores with each unit increase in age (B = .19, P = .02). Moreover, the model predicted more than a five-point decrease in the lifestyle behavior change scores among participants reporting a gain in weight or were unsure about the weight gain (B = −5.56, p < .001). These findings have significant implications for healthcare providers and policymakers. Actions to promote healthy lifestyle behaviors are essential to battle the increased risks of obesity after the pandemic, particularly among young adults. Additionally, mental and psychological wellbeing support during crises are vital for maintaining healthy lifestyle choices.

## 1 Introduction

The World Health Organization (WHO) announced a global Coronavirus Disease (COVID-19) pandemic in January 2020. As of August 2022, the virus has infected over 579 million individuals and resulted in more than six million deaths globally [1]. Most countries instituted preventative measures to combat the rapid spread of COVID-19, including isolation, social distancing, and lockdowns. Although these pandemic preventive measures largely limited the spread of the infection, they have forced changes in the lifestyle of individuals [2].

In Oman, the government implemented general preventive measures, including wearing masks, avoiding hand contact, limiting official and social gatherings, closing educational institutions, and quarantining or isolating at-risk or affected individuals [3]. These measures were in place from the beginning of 2020 until mid-2022 [4], which could have led to deviations in lifestyle behaviors for an extended period. There were reports of worsened eating habits among school-age children, including skipping breakfast and reduced fruit and vegetable consumption [5], but the impact on the adult population remains unclear.

The global literature illustrates that the lifestyle changes brought about by the pandemic have resulted in diverse outcomes. A scoping review of 23 papers representing adult populations from the United States, Europe, Africa, Asia, and Australia revealed mixed outcomes related to changes in dietary patterns during the pandemic. While most reported adverse nutritional outcomes such as increased snacking and meal frequency, others demonstrated favorable shifts by consuming fresh and homemade food [6]. Another review of studies from 17 Middle Eastern countries presented comparable proportions of improvement or worsening nutritional habits, 30.9 % versus 24.8 %, respectively [7].

The contradicting findings presented by these reviews limit the consensus of the pandemic's effect on people eating behaviors. That said, a systematic review of 23 longitudinal international studies conducted across the globe compared changes in eating behavior before and after the pandemic. The results indicated a general trend toward deteriorating eating behaviors, reflecting increased snacking frequency and a preference for sugary and heavily processed foods over fruits and vegetables [8]. Besides, data from Europe, the Americas, Asia, Africa, and Australia showed an overall decrease in physical activity levels and an increase in sedentary behaviors [9]. Similarly, a review from the Middle East reported noticeable rates of physical inactivity and sedentary behaviors [7]. These adverse lifestyle behavior outcomes have not only imposed risks to the individual's physical health but were associated with disrupted mental well-being [10]. This has added burden on the healthcare system with unblemished influence on the population's mental health with variant levels of stress, anxiety, and depression [11].

Various community-related factors, including cultural background, pre-pandemic lifestyle norms, and access to healthcare resources, could moderate lifestyle behaviors during the pandemic, resulting in distinct outcomes from country to country. To address specific emerging needs resulting from the pandemic, each nation should carefully analyze its population's distinct effects of the pandemic and develop tailored interventions and support mechanisms. Notably, further research is needed to understand the changes in lifestyle behaviors among adults in Oman during the pandemic.

Despite the tremendous amount of literature on COVID-19's impact on lifestyle behaviors, there remains a gap related to the interplay between pandemic preventative measures, sociodemographic characteristics, lifestyle changes, and health consequences. This is particularly true for selected populations like Oman, which already has high rates of metabolic diseases and obesity. Oman has struggled with increasing rates of metabolic problems before the COVID-19 pandemic. According to a recent national survey, 66 % of Omani adults are overweight or obese, and cardiovascular diseases and diabetes account for 36 % and 8 % of non-communicable deaths in Oman, respectively [12]. Nonetheless, a comprehensive study has yet to adequately explore the sociodemographic factors associated with the impact of the pandemic on lifestyle-related habits and whether these changes have contributed to an increased risk of metabolic disorders.

This study examined the sociodemographic and health characteristics determinants of lifestyle behavior changes among Omani adults during the COVID-19 pandemic. Sociodemographic factors investigated were age, gender, education, study (college versus non-college students), and marital status. Health-related factors studied were a history of COVID-19 infection and a change in weight during the pandemic. The results of this study have the potential to benefit the population in Oman and contribute to the global understanding of the pandemic's multifaceted impacts on lifestyle behaviors and health outcomes. Furthermore, tailored interventions and support systems could be developed based on insights generated from this study and serve as a model for addressing the specific needs due to the pandemic in other nations.

## 2 Materials and methods

### 2.1 Research design

This study followed a cross-sectional research design. This design was suitable for collecting general data about how COVID-19 has influenced people's lifestyles and was used previously in similar international studies [2].

### 2.2 Population and setting

We recruited participants using a convenient sampling method. Convenience sampling was utilized in this study to facilitate recruiting adult participants from the general population. Given the research's descriptive nature and limited resources, this method was deemed suitable [13]. Based on a confidence level of 95 %, a margin of error of within ±5 %, and a population proportion of 50 %, the minimum sample size required is 385. Inclusion criteria included being an adult (18 years or older), Omani, and agreeing to participate in the study. Children and adolescents younger than 18 years were excluded. Data were collected in Oman between January and March 2022 using the Google online survey platform. An online Google questionnaire was distributed to adults via social media, including WhatsApp, Twitter, and Instagram.

### 2.3 Study instruments

We collected information related to sociodemographic and health-related characteristics. Participants were asked about their age, gender, educational level, study status, and marital status. Additionally, we asked participants if they had a history of COVID-19 infection (no, yes, suspected) and weight changes during the pandemic (stable, lost, gained, unsure).

We adopted the Impact of COVID-19 on Lifestyle-related Behaviors questionnaire [14] to assess the lifestyle behavior changes during the pandemic. The questionnaire included 20 items that measured different lifestyle behaviors, including dietary habits (14 items), physical activity (3 items), sleep patterns (2 items), and stress and anxiety (1 item). The dietary habits items (# 1–14) ask about the patterns, size, and frequency of meals, food groups, consumption of food high in sugar, fat, and salt, sugar-sweetened drinks, emotional eating, changes in cooking, as well as consumption of foods that enhance the immune system. The physical activity items (# 15–17) are related to aerobic exercise, housework, leisure activity, sitting, and screen time. Sleep pattern items (# 18, 19) focus on the amount and quality of sleep, while the stress and anxiety item (# 20) asks about the change in the level of anxiety and stress.

The questionnaire is answered on a 5-point Likert scale (i.e., significantly increased, slightly increased, grossly similar, slightly decreased, significantly decreased) [14]. The scoring for items "1″, "2″, "6″, "7″, "8″, "9″, "10″, "17″, and "20″ were as follows: 2 = significantly increased, −1 = slightly increased, 0 = grossly similar, 1 = slightly decreased, 2 = significantly decreased. The scores for items "4″, "5″, "11″, "12″, "13″, "14″, "15″, "16″, and "19″ were reversed as follows: 2 = significantly decreased, −1 = slightly decreased, 0 = grossly similar, 1 = slightly increased, 2 = significantly increased. The scores for items 3 and 18 were: 2 = significantly decreased/increased, −1 = slightly decreased/increased, and 0 = grossly similar" [14]. The sum score ranges from −40 to 40. There is no cut score for healthy versus unhealthy behaviors. However, a higher total score indicates healthier lifestyle changes and vice versa. The questionnaire underwent an intensive development process that involved a literature review, focused group discussions, expert evaluation, and pre-testing. The Cronbach's alpha was .72, and a group of five experts in medicine, nutrition, endocrinology, metabolism, and clinical psychology established the questionnaire's face and content validity [15].

### 2.4 Pilot study

The authors granted permission from the questionnaire developer, Dr. Piyush Ranjan, to use and translate the questionnaire into Arabic. The questionnaire was back-translated from English to Arabic and Arabic to English by a professional translator. Then, the translation was further validated by a bilingual expert in healthy lifestyle research. The Arabic version of the questionnaire was then pilot-tested among 20 adults for reliability and clarity. The participants were asked to complete the questionnaire, assess each item's level of comprehension and relevance, and recommend any modifications. The responses suggested adequate clarity of the questionnaire, and no suggestions for modification were received. Therefore, no changes were applied to the translated questionnaire. The Cronbach alpha of the translated questionnaire was .67, probably due to the small sample size, which is considered acceptable [16]. Data from the pilot study were not included in the final study analysis.

### 2.5 Data collection procedures

We invited participants through messages on social media platforms, including WhatsApp, Instagram, Twitter, and Snapchat. The invitation message included a link to the study questionnaire for the participants willing to participate. Social media is a convenient way to have a large and diverse group of participants. In addition, it is a cost-effective, time-saving, and resource-saving strategy that improves community engagement [17].

The purpose of the study, benefits, duration of the questionnaire, confidentiality, and participants' rights were all explained in the study description section. Participants were informed that participating in the study is voluntary and that participants may withdraw from the study with no consequences. Researchers' contact details for inquiries were shared in the electronic invitation message. An agreement statement was included at the beginning of the questionnaire, and participants were required to select an option before moving forward, either "I agree" or "I do not agree." All participants who completed the questionnaire chose the "I agree" option. The study data were anonymous as no identification information such as name, date of birth, or medical record number was asked. All participant data were maintained in a password-protected computer, and only the research team could access the data.

## 4 Results

### 4.1 General characteristics of participants

A group of 515 participants, with an average age of 25.7 years (SD ± 6.83) and a majority of 73.4 % females, contributed to our research. Furthermore, 58.3 % of the participants held at least a bachelor's degree and 45.4 % were college students. Among respondents, 59.8 % had a positive or suspected history of COVID-19 infection. In addition, 46.2 % reported gaining weight or were unsure during the pandemic.

### 4.2 Description of changes in lifestyle behaviors

Table 1 shows the self-reported dietary habits, physical activity, sleep, stress, and anxiety changes during the COVID-19 pandemic. Regarding lifestyle behaviors related to dietary habits, descending negative changes were observed in the portions of meals and snacks, cooking new/traditional recipes, snacking between meals, consumption of unhealthy food when bored, stressed, or upset, skipping the main meals, consumption of sweets/candies/chocolate, and consumption of junk food/fast food and fried food. On the other hand, higher to lower positive changes were observed in supporting family and friends eating healthy food, intake of immunity-boosting foods, interest in learning healthy eating tips from the media, intake of nutrition supplements to boost immunity, daily intake of fruits and vegetables, intake of sugar-sweetened beverages, and intake of a balanced diet.

**Table 1:** Means, medians, and standard deviations of changes in lifestyle behavior during COVID-19.

	**Mean**	**Median**	**SD**
***Dietary Habits***			
Item 1. Skipping one of the main meals (breakfast/lunch/dinner).	-.29	0	.99
Item 2. Snacking between meals.	-.56	−1	1.08
Item 3. Quantity/portions of meals and snacks.	-.77	−1	.71
Item 4. Daily intake of fruits and vegetables.	.15	0	1.07
Item 5. Intake of a balanced diet.	.03	0	.93
Item 6. Consumption of junk food/fast food and fried food.	-.11	0	1.31
Item 7. Intake of sugar-sweetened beverages.	.04	0	1.27
Item 8. Consumption of sweets/candies/chocolate.	-.15	0	1.19
Item 9. Participation in cooking new/traditional recipes.	-.72	−1	1.00
Item 10. Consumption of unhealthy food when you are bored, stressed, or upset.	-.56	−1	1.08
Item 11. Intake of immunity-boosting foods.	.45	0	1.01
Item 12. Intake of nutrition supplements to boost immunity.	.24	0	.84
Item 13. Support of your family and friends in eating healthy.	.53	0	.99
Item 14. Interest in learning healthy eating tips from the media.	.45	0	.93
***Physical Activity***			
Item 15. Participation in aerobic exercise.	-.05	0	1.16
Item 16. Participation in leisure and household chores.	.63	1	1.11
Item 17. Sitting and screen time.	-.44	−1	1.21
***Sleep***			
Item 18. Hours of sleep.	-.94	−1	.74
Item 19. Quality of sleep.	-.07	0	1.05
***Stress and Anxiety***			
Item 20. Stress and anxiety levels.	-.59	−1	.98

Moreover, results showed negative changes related to sitting and screen time and participation in aerobic exercise. However, there were positive changes relevant to participation in leisure and household chores. Regarding sleep, stress, and anxiety, negative changes were observed across hours of sleep, stress and anxiety levels, and quality of sleep, respectively.

### 4.3 Associations of changes in lifestyle behaviors

In this study, the total lifestyle behavior change mean score was -2.75 (SD ± 9.08), while the median was -2.00. The score ranged from −37 to 25. Table 2 describes the sociodemographic and health characteristics of the participants, the respective lifestyle behavior change means, and their bivariate association. Pearson's correlation test showed that lower lifestyle behavior change scores (unhealthier lifestyles) were associated with younger age (p < .001). Besides, independent *t*-test analysis revealed that lifestyle behavior change scores were associated with being single (p = .012), being a college student (p = .004), and having gained weight or were unsure about the weight change during the pandemic (p < .001).

**Table 2:** Association between sociodemographic and health characteristics with the mean of lifestyle behavior change (LCS) score.

	**Frequency (%)/Mean (SD)**	**LCS Mean (SD)/Pearson correlation**	**P value**
Age		.	.000
Mean (SD)	25.7 (6.83)	16	
Gender			.903
Male	136 (26.4)	−2.67 (7.85)	
Female	378 (73.4)	−2.78 (9.50)	
Educational level			.627
Less than a bachelor's degree	215 (41.7)	−2.52 (9.12)	
Bachelor's degree or more	300 (58.3)	−2.92 (9.05)	
Marital status			.012
Single	322 (62.5)	−3.53 (8.36)	
Married	193 (37.5)	−1.45 (10.04)	
Study Status			.004
College student	234 (45.4)	−4.03 (8.85)	
Non-college student (job seeker/employee/free business/housewife/retired)	281 (54.6)	−1.69 (9.14)	
History of COVID-19 infection			.398
No	207 (40.2)	−2.34 (10.17)	
Yes/suspected	308 (59.8)	−3.03 (8.26)	
Weight change during COVID-19			.000
Stable/lost	277 (53.8)	−.22 (8.01)	
Gained/unsure	238 (46.2)	−5.69 (9.37)	

### 4.4 Linear regression analysis of changes in lifestyle behaviors

Table 3 shows the results of a linear regression model. The variables that remained independently associated with an unhealthier lifestyle (i.e., lower scores) were a younger age and weight gain or unsure during the pandemic. The model predicted a .19 increase in the lifestyle behavior change scores (more positive change) with each unit increase in age (B = .19, p = .02). Additionally, the model predicted more than a five-point decrease in the lifestyle behavior change scores (more negative change) among the group reporting gain in weight or was unsure about the weight gain during COVID-19 (B = −5.56, p < .001).

**Table 3:** Linear regression analysis of changes in lifestyle behavior during COVID-19.

	**B**	**Std. Error**	**t**	**P value**
Age	.19	.08	2.33	.020
Study Status	.47	1.08	.44	.662
Marital Status	.18	1.18	.16	.877
Weight Change	−5.56	.76	−7.32	.000

Note. Dependent is the sum score of changes in lifestyle behaviors during COVID-19.

## 5 Discussion

This study has investigated the sociodemographic and health characteristics associated with lifestyle behavior changes among Omani adults during the COVID-19 pandemic. The sociodemographic factors investigated were age, gender, education, study, and marital status. Health-related factors considered were a history of COVID-19 infection and a change in weight during the pandemic.

According to this study, Omani adults generally presented with negative dietary habits, physical activity, sleep stress, and anxiety outcomes during the COVID-19 pandemic. This finding is consistent with the global literature. A systematic review of 23 longitudinal studies reported increased snacking habits during the COVID-19 pandemic [8]. Likewise, a scoping review manuscript reported increased unhealthy food consumption, such as desserts, fried food, and processed foods [6]. On the other hand, a longitudinal study conducted in Italy found deceased junk and fast food intake during the pandemic [18]. Also, a Russian study reported reduced consumption of unhealthy eating behaviors such as snacking and consumption of pastries [19].

The contradicting outcomes could be related to differences in governmental policies instituted during the pandemic on the fast-food restaurant market. For instance, restaurant and food markets in Oman remained active during the pandemic through pick-up and home delivery. This may have facilitated increased frequency of snaking as COVID-19 infection control regulations required people to stay home, and frequent snacking is usually associated with boredom [20]. However, in other parts of the world, such as Russia, the restaurant and catering industry shrank 24.5 % between January and July 2020 compared to 2019 [21]. The healthier dietary behaviors during the pandemic could also be attributed to people's vigilance in boosting their immunity with nutrition-rich food choices, especially since they spent most of their time at home and could cook fresh meals. Additionally, the differences in the dietary outcomes could be moderated by various social and environmental factors such as pre-pandemic lifestyle and cultural norms, as well as access to healthcare resources, which warrants further investigation.

Our study exhibited a noticeable leisure and household chores increase during the pandemic. Increased sitting time and time spent in front of screens during the pandemic was consistently reported in global literature [22]. Besides, there is a consensus among studies from around the world on decreased physical activity during the pandemic [23]. Spending more time at home due to self-isolation and lockdowns had general adverse effects on the physical activity level. There is an agreement that pandemic protection measures, including self-isolation, lockdown, and governmental policies, although contributed to decreased infection rates, have limited the opportunities for the population to exercise in gyms or public places [24].

Our study findings also indicated disturbed sleeping patterns, mainly in the hours of sleep. A large cross-sectional study (N = 2137) from Iraq reported a significant increase in sleeping hours during lockdown [25]. Disturbed sleeping quality could be attributed to the pandemic-associated stress. A German study indicated a higher stress level during the pandemic predicted lower sleep quality [26]. Notably, and similar to our study, several studies conducted in Oman reported intensified psychological stress and altered quality of life during COVID-19 [[27], [28], [29]], which explains the tendency toward disturbed sleeping patterns, snacking, junk food consumption, and poor drive for physical activity [30]. These negative experiences and anxiety associated with the daily pandemic news have potentially resulted in body physiologic stress reactions that mimic the satiety sensation induced by feeding and the feeling of less energy [31]. A multicenter study representing five Ibero-American Countries found that less healthy eating pattern during the pandemic was associated with anxiety and a positive history of COVID-19 infection [32].

According to our findings, changes in lifestyle-related behaviors during the COVID-19 pandemic were prominent among younger adults. However, this finding should be interpreted cautiously, as it could result from the sample's predominantly young adult composition. That being said, adverse changes in lifestyle-related behaviors among young adults during the COVID-19 pandemic are consistent with the global literature [33,34]. Furthermore, the regional literature reported similar findings. A study conducted in the United Arab Emirates found that younger adults were more prone to practice unhealthy lifestyle-related behaviors during the pandemic than older adults [35]. Additionally, it was observed that COVID-19 preventive measures have adversely affected the physical fitness levels of the general population, especially young adults [36]. On the contrary, a study from Sicily found that older adults were more affected by negative physical activity behaviors during the pandemic [37]. In general, older people are more committed and tend to maintain their habits, particularly nutritional habits, even during unexpected circumstances, as opposed to the younger population [38]. Furthermore, younger people are at a higher risk of experiencing emotional distress as they are often single college students and, therefore, lack the same level of emotional support as older adults [39,40]. Consequently, they are more likely to change lifestyle behaviors.

In our study, more negative lifestyle behavior changes were reported among participants who experienced gaining weight during the pandemic. Multiple studies conducted during the pandemic support these findings. A cross-sectional study conducted in the Chilean national territory among 700 participants found that increased weight during the pandemic positively correlated with eating fried food more frequently, drinking less water, and becoming less active [41]. Moreover, a longitudinal study conducted in the United States during the pandemic reported that the participants who gained weight were more exposed to unhealthy dietary intake and reduced physical activity performance [42]. This may indicate a potentially health-related outcome to the reported negative lifestyle behaviors. Moreover, the duration of the pandemic preventive measures, almost two years, contributed to a prolonged effect on lifestyle behaviors, ultimately resulting in weight status changes [43].

Unfortunately, an increase in obesity rates was reported during the pandemic, which augmented the burden of metabolic diseases across the globe. A systematic scoping review reported that half of the adult population had gained weight during the pandemic [44]. Similarly, a retrospective study conducted in China showed a significant increase in average BMI during the pandemic [22]. Moreover, the gain in weight was more observed among women [45]. However, our study revealed that gender did not determine behavior change during the pandemic, showing an equal likelihood of unhealthy lifestyle behaviors and risk of obesity across the genders.

Furthermore, higher or lower educational levels were not associated with Omani adults' lifestyle changes. The literature reported consistent findings [34]. Higher educational status was not necessarily a protective factor for healthy lifestyle behaviors in Oman and the region [46,47].

The reported unfavorable lifestyle habits underscore the urgency of understanding the pandemic's impacts. These habits have the potential to elevate susceptibility to non-communicable diseases such as obesity, type 2 diabetes, and cardiovascular diseases. Expanding our understanding of the pandemic's multifaceted impacts, we can develop individualized interventions to support population health and wellbeing. Through this understanding, we can mitigate negative effects and promote positive behavior changes, ultimately contributing to the overall wellbeing of the population in Oman and beyond. This work stands out for its unique and comprehensive approach to investigating the complex interplay between the pandemic and lifestyle behaviors and the associations with sociodemographic factors, history of COVID-19 infection, and risk for obesity.

This study has a few limitations. We utilized a cross-sectional design, and participants self-disclosed their infection history and weight changes, potentially introducing some biases. Additionally, the convenience sampling technique resulted in the sample being predominantly composed of young adult women, which limits the generalizability of findings. However, collected data has provided insights into the effect of such pandemic-related restrictions on people's general health, which lays the foundation for future research studies and preventive programs.

### 5.1 Implications

The COVID-19 pandemic has negatively affected lifestyle-related behaviors among the Omani adult population, resulting in increased weight gain. Therefore, public health sectors may invest further in obesity management, probably through lifestyle behavioral interventions. In any future compelling situation, such as the COVID-19 pandemic, governments should be prepared for attainable actions to promote adherence to healthy nutritional intake, physical activity, sleeping practices, and maintaining healthy weight statuses, particularly among young adults. In addition, mental and psychological wellbeing services would be accessible during crises to counteract any further deterioration of healthy lifestyle choices.

Future researchers may consider using longitudinal study designs to establish the causality between pandemic-associated lifestyle behavior changes and sociodemographic characteristics. Additionally, using objective anthropometric measurements in the future would strengthen the conclusion related to crises influence on weight status.

## 6 Conclusion

Overall, this study provides information necessary to counteract the impact of COVID-19 or any future compelling situation on the population's lifestyle-related behaviors. The results showed a negative impact of the COVID-19 pandemic on lifestyle-related behaviors, particularly among young adults. As expected, people who gained weight during the pandemic had more negative changes in their lifestyle patterns. The COVID-19 pandemic seems to worsen the rates of obesity worldwide. Therefore, public health sectors should invest further in obesity management, probably through lifestyle behavioral interventions. Future research may benefit from using longitudinal designs and objective measurements.

## CRediT authorship contribution statement

**Basma Al Yazeedi:** Writing – review & editing, Validation, Supervision, Project administration, Methodology, Formal analysis, Conceptualization. **Azza Al Marshoudi:** Writing – original draft, Project administration, Methodology, Investigation, Data curation, Conceptualization. **Hajar Alufi:** Writing – original draft, Methodology, Investigation, Formal analysis, Data curation, Conceptualization. **Mallak Al Salmi:** Writing – original draft, Visualization, Validation, Investigation. **Dakariyat Al-Sharji:** Writing – original draft, Visualization, Validation, Investigation. **Yaqoob Al Hinai:** Writing – original draft, Methodology, Investigation.

## 3 Ethical statement

The study was ethically approved by the College of Nursing Research and Ethics Committee (CON/GP/2022/10). The purpose of the study, benefits, inclusion criteria, duration of the questionnaire, confidentiality, and participants' rights were explained in the invitation message. Participants were informed that participating in the study is voluntary and that they may withdraw from the study with no consequences. Researchers' contact details for inquiries were shared in the electronic invitation message. A consent statement was included at the beginning of the questionnaire, and participants were required to select an option before moving forward, either "I agree to participate in the study" or "I do not agree to participate in the study." The study data were anonymous as no identifying information such as name, date of birth, or medical record number was asked. All participant data were maintained in a password-protected computer inside a locked office, and only the research team had access to the data.

### 3.1 Data analysis

We thoroughly analyzed the study data using the Statistical Package for Social Sciences (SPSS) version 24.0. Our analysis began with determining descriptive statistics, including mean, standard deviation, frequency, and percentages. To establish the association between the total score of lifestyle behavior change and age, we employed Pearson's correlation test. We then conducted an independent *t*-test to assess the relationship between the lifestyle behavior change score and various factors such as gender, education, marital and study status, history of COVID-19 infection, and change in weight during the pandemic. To ensure the accuracy of our analysis, we converted these factors into dichotomous variables. Finally, we used linear regression analysis to identify the relationship between the lifestyle behavior change score and the factors that showed a significant relationship.

## Data and code availability statement

Data will be made available on request.

## Declaration of competing interest

The authors declare that they have no known competing financial interests or personal relationships that could have appeared to influence the work reported in this paper.
